# A research on syndrome element differentiation based on phenomenology and mathematical method

**DOI:** 10.1186/s13020-017-0141-1

**Published:** 2017-07-10

**Authors:** Enliang Yan, Jialin Song, Chaonan Liu, Wenxue Hong

**Affiliations:** 1 Institute of Electrical Engineering, Yanshan University, No. 438, Hebei Avenue, Qinhuangdao, 066004 Hebei People’s Republic of China; 2 Guangzhou University of Chinese Medicine, Guangzhou, Guangdong 510405 People’s Republic of China

**Keywords:** Syndrome element, Syndrome differentiation, Traditional Chinese medicine, Mathematical mapping, Phenomenology, Attribute partial-ordered structure diagram

## Abstract

**Background:**

As an empirical medical system independent of conventional Western medicine (CWM), over thousands of years, traditional Chinese medicine (TCM) has established its own unique method of diagnosis and treatment. The perspective of holism and system in TCM is essentially different from the view of Reductionism in CWM. With the development of modern science and technology, the restriction of reductionism is more and more prominent, and researchers begin to pay more attention to holistic thinking in TCM. Confronted with the above situation, there is an urgent need to explore the diagnosis of TCM by the techniques of modern science.

**Methods:**

To explore the feasibility of using modern science to describe and realize the diagnosis of TCM, in this paper, a method of syndrome element differentiation based on phenomenology is proposed. The proposed method is implemented by mathematical mapping, and then it is testified through analysis of 670 medical records: Based on the original mapping data between two data sets (set of syndrome elements and set of clinical manifestations), new mapping data is generated, and thus the corresponding quantitative diagnostic results are calculated and evaluated. Finally, knowledge discovery of the diagnosis results based on attribute partial-ordered structure diagram is conducted.

**Results:**

The value order’s matching results between original and new results show that the matched degree of each record is no less than 65%, while there are at least 87% records whose matched degree is more than 80%. In addition, the knowledge discoveries of new results are basically identical with the ones of original results as well.

**Conclusion:**

Using phenomenology to describe syndrome differentiation should be feasible, and further research on mapping relations between various sets (symptoms, formulas, drugs) of TCM should be conducted and evaluated through clinical trials in future.

**Electronic supplementary material:**

The online version of this article (doi:10.1186/s13020-017-0141-1) contains supplementary material, which is available to authorized users.

## Background

With the development of science and the change of living environment, people’s cognition of health has deepened, and the focus of medical science has gradually shifted from disease treatment to prevention and healthcare. Therefore, it has been an urgent issue to evaluate health state objectively and accurately. As an important and irreplaceable constitution of modern medicine, over the past decades, traditional Chinese medicine (TCM) has gained wide attention in the medical field of both domestic and abroad. In terms of both theory and practice, TCM provides an essentially distinct medical approach compared to conventional Western medicine (CWM). Taking holism as core, TCM has unique advantages in the aspects of health maintenance and disease prevention. Meanwhile, with the dramatic increase in prevalence of chronic diseases, the treatment of CWM has begun to be stretched, while the natural medicine and therapy of TCM can contribute a lot to this condition. Therefore, TCM has attracted unprecedented expectations and attention [1]. However, despite the great advantages, the understanding, education and application of TCM is relatively insufficient, the main reason of which may be that the diagnosis of TCM is equivocal in the perspective of modern science. Therefore, using methods of modern science to describe and realize the diagnosis of TCM has been an urgent issue.

Over the past decades, with the goal of modernization, research on TCM diagnosis has attracted significant attention. Wang constructed a quantitative system for pulse diagnosis [2] and proposed a quantitative method for syndrome differentiation [3] based on Bayesian networks. Wang also proposed a method based on decision tree to explore the quantitative recognition of pulse strength [4]. To make both qualitative and quantitative analysis for analysis for facial complexion, Zhao [5] proposed a feature representation of facial complexion from whole face of patients. Using multi-class support vector machine, Li [6] designed a computer-assisted classification method for syndrome diagnosis based on lip images. Liu [7] explored a multi-label learning technique to do inquiry diagnosis for CHD in TCM. Su [8] reviewed the technologies and methods and their application in syndrome differentiation for TCM.

These studies provide valuable experience and guidance for the research of syndrome differentiation in TCM. However, even a large amount of TCM diagnosis system is developed by computational methods, and most of them claimed that their methods or systems could analyze TCM data from a quantitative perspective. Actually none of them could quantize their diagnostic data with meaningful implications corresponding to TCM theory, as the clinical indicators from the perspective of CWM. If this situation could not be improved, the establishment of quantitative diagnosis of TCM may be very difficult [9]. As was Prof. Qian said, the theory of TCM is not natural science, while it is natural philosophy which is based on phenomenological cognition [10], that’s why the classical methods for CWM are not suitable for TCM. Therefore, it is still a challenging issue to develop an approach which can both realize the quantitative diagnosis of TCM in modern science and be consistent with the phenomenological cognition of TCM.

In view of the above situation, to explore the feasibility of using modern science to describe and realize the diagnosis of TCM, a method of syndrome differentiation, which is based on phenomenology TCM, is proposed in this article. The approach can realize the quantitative diagnosis of TCM, and it is implemented by mathematical mapping.

This paper is organized as follows: “Background” introduces the research background and the motivation of the study. “Theories” describes the theories adopted in this paper, including phenomenology, syndrome element differentiation and attribute partial-ordered structure diagram. “Methods” explains the methods of clinical data acquisition, mapping data reconstruction, matching, evaluation and knowledge discovery of results. “Results” shows the results of data processing and knowledge discovery. “Discussion” discusses the results of the research. “Conclusion” draws the conclusion of the study.

## Theories

### Theory of phenomenology

Phenomenology, proposed by philosopher Edmund Husserl, is a philosophical methodology [11]. Phenomenological researchers believe that people usually cognitive the world through direct experience and ideological processing, which is called ‘phenomenological method’ in the field of physics. In the perception of phenomenology, the microscopic cause of phenomenon is not so important, while associations between diverse phenomena are the key points, and these associations can be acquired by summing up experience and summarizing experimental facts.

To sum up, concentrating on the research of ‘phenomena’: appearances of things, or things as they appear in our experience, or the ways we experience things [12], phenomenology refers to the system theory which analyzes, induces and summarizes the essence of things by the phenomenon, which happens to be consistent with the thought of TCM. TCM is also a qualitative theory which uses a summarization of the associations between phenomena or functions, not detail description of concrete mechanism [13]. Therefore, using phenomenology to describe the diagnosis of TCM should be feasible in theory.

**Fig. 1:**
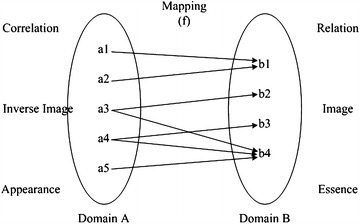
Mathematical description of phenomenological theory

Figure 1 shows the mathematical description of phenomenology. As shown in the figure, the appearance of things can be regarded as a source domain set, while the essence of things can be seen as an image domain set, and the relations between appearance and essence can be described by generalized mapping. As philosophers say, our conception (phenomenon) of natural laws (mapping) depend on our approach to understanding reality (essence), there is no theory-independent concept of reality, and every law (mapping) we acquired is only an approximation of reality. In real life, the approximation of mapping between appearance and essence can be acquired by observation, induction, deduction and many other kinds of machine learning methods.

### Theory of syndrome element differentiation

As a peculiar rational concept in TCM, syndrome is the combination of philosophy, epistemology, medical theory and clinical practice. It is a physiological or pathological generalization of the overall health state of a body at a given stage. Syndrome usually consists of two parts: location and essence. Syndrome differentiation is the process of obtaining the location and essence of syndrome through overall analysis of clinical manifestations acquired from patient by four examinations and achieving a syndrome name which can represents the health state of the patient. In TCM, for the diagnosis and treatment of disease, it is essential to identify the syndrome accurately and precisely [14].

Syndrome element differentiation is a method of syndrome differentiation proposed by Prof. Zhu [15], and in his theory, the process of syndrome differentiation is divided into two parts: quantification of syndrome elements according to clinical manifestations and syndrome matching based on the quantification of syndrome elements [16].

**Fig. 2:**
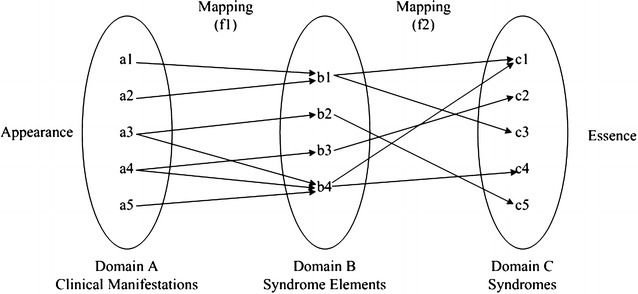
Mathematical description of syndrome element differentiation

Figure 2 shows the mathematical description of syndrome element differentiation. From the perspective of phenomenology, the process of syndrome element differentiation can be regarded as two mappings between three domain sets, and the key to syndrome differentiation is to discover these two mapping relations.

With years of research, extracting from classical literature and clinical records, Prof. Zhu has given out the original mapping data between clinical manifestations and syndrome elements [16].

### Theory of attribute partial-ordered structure diagram (APOSD)

APOSD, which can extract knowledge from formal context and visualize the results in intelligible diagram, is a method of knowledge discovery proposed by Prof. Hong [17]. APOSD stems from formal concept analysis (FCA), partial order of mathematics is its basis of data analyzing and the generation of APOSD is identical with the philosophical principle of human cognition of things [18].

Formal context is the data basis of APOSD. A formal context $\varvec{K = (U, M, I)}$ consists of two sets $\varvec{U = \{ u1,u2, \ldots ,un\} }$ and $\varvec{M = \{ m1,m2, \ldots ,mk\} }$ and a relation ***I*** between ***U*** and ***M***. The elements of ***U*** are called the objects and the elements of ***M*** are called the attributes of the context. The data shown in Table 1 is a classical example of formal context.

**Table 1:** Formal context of example

	a1	a2	a3	a4	a5	a6	a7	a8	a9
o1	1	1					1		
o2	1	1					1	1	
o3	1	1	1				1	1	
o4	1		1				1	1	1
o5	1	1		1		1			
o6	1	1	1	1		1			
o7	1		1	1	1				
o8	1		1	1		1			

As shown in Table 1, the data in the first row is the set of attributes, while the data in the first column is the set of objects, and the number ‘**1**’ in the intersection of object and attribute means the object has the attribute, or the attribute belongs to the object.

**Fig. 3:**
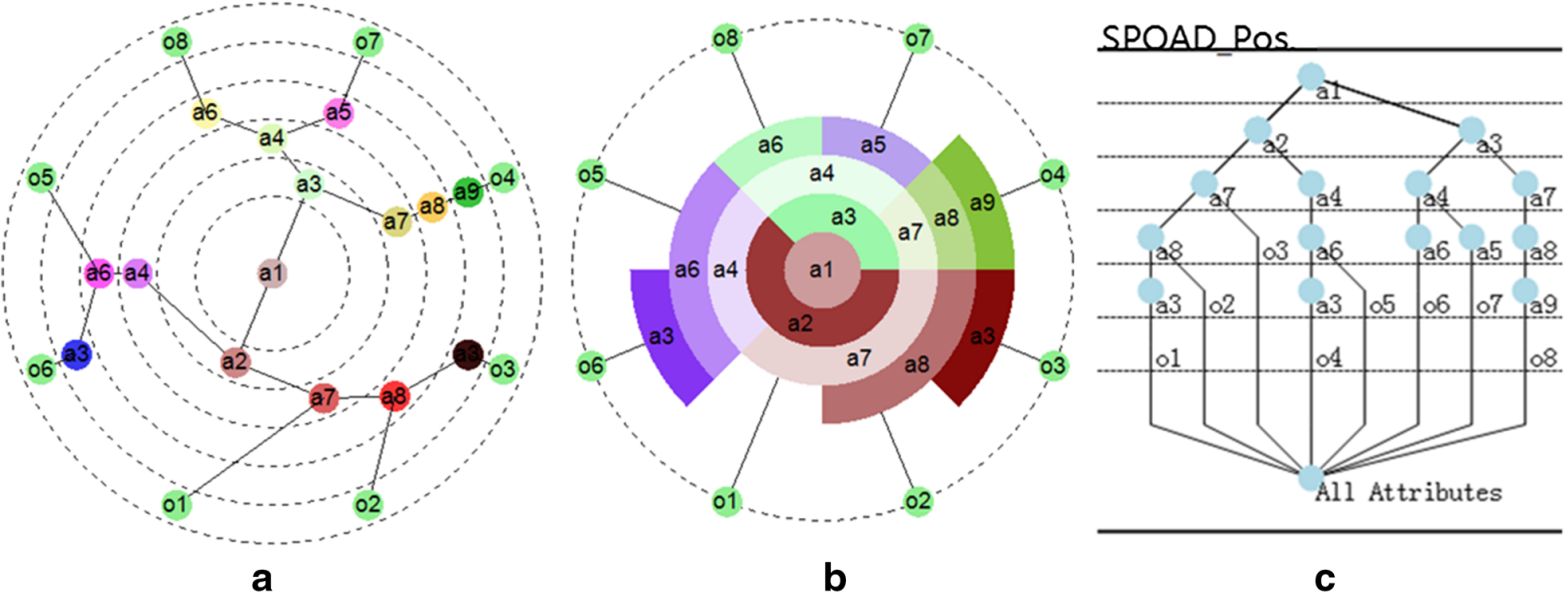
APOSD of biology and water.** a** Star style,** b** Annular style,** c** Tree style

Similar to formal concept analysis (FCA), APOSD emphasizes cognitive ability and concentrates on the relation between different data sets. The difference between FCA and APOSD is that FCA focuses on the generation and analysis of concept and concept lattice, while APOSD concentrates on the study of attributes’ feature. Based on the formal context in Table 1, using the definition of attributes’ feature [19] and the method of data processing [17], the APOSDs shown in Fig. 3 are generated.

As shown in Fig. 3, APOSD can be presented in three styles: star [20], annular and tree. In APOSD, sequential structure visualization model is adopted. From top to bottom (tree style), or from inner to outer (star and annular style), the nodes of attributes represent the constitution of the corresponding object and the layer each attribute node located in shows the universal degree of the attribute. The attribute located in the innermost (toppest) layer has the highest university (covering the most objects).

Over the past decade, APOSD has been widely employed in the knowledge discovery for TCM, and it proved effective in the field of TCM [21–24]. Therefore, APOSD is adopted to analyze the combination structure of syndrome elements based on the quantitative results of 670 medical records.

## Methods

The minimum standards of reporting checklist contains details of the experimental design, and statistics, and resources used in this study (Additional file 1).

### Acquisition of clinical records

#### System design of data acquisition

**Fig. 4:**
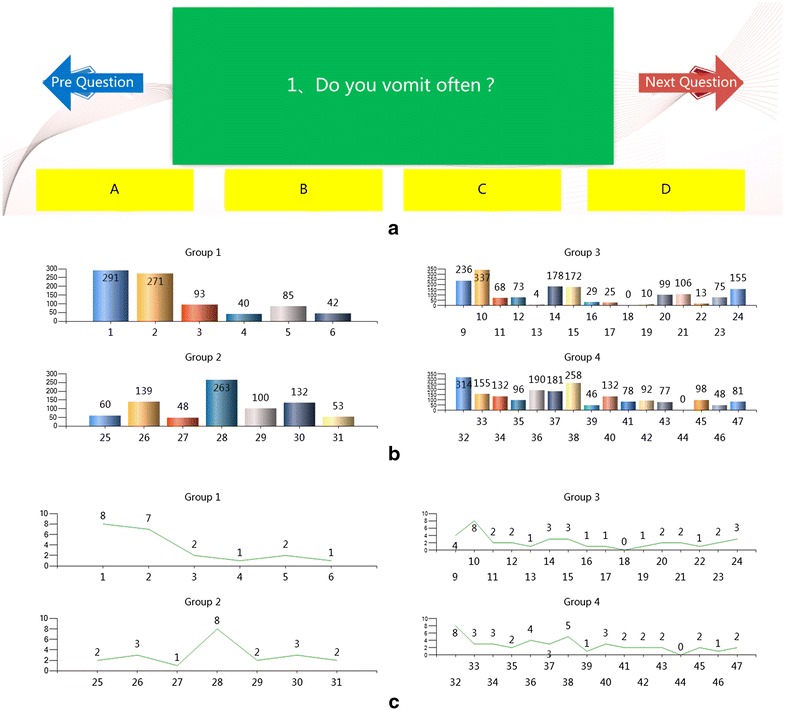
System of data acquisition.** a** Question of inquiry,** b** value of syndrome elements,** c** value orders of syndrome elements

Based on the theory of syndrome element differentiation, with the support of National Science Foundation of China (NSFC, No. 61074130), a prototype system (Fig. 4) of syndrome element measurement was designed by the team of Prof. Hong [25].

In the system, for the clinical manifestations mapping to syndrome elements, 177 inquiry questions (related to symptoms or signs common in clinical diagnosis) are designed for male, while 194 inquiry questions are designed for female. According to the severity and frequency of each symptom or sign, the answer to the corresponding question can be divided into four levels (Fig. 4a; Table 2).

**Table 2:** List of input answers

No	Label	Severity	Frequency	Weight
5	A	Severe	Always	1
4	B	A little severe	Sometimes	0.618
3	C	Not severe	Seldom	0.382
0	D	None	Never	0

The output quantitative values (Fig. 4b) and value orders (Fig. 4c) of 47 syndrome elements (divided into four groups, Table 3) are acquired from clinical input data (answers to symptoms and signs) and the corresponding mapping data between syndrome elements and clinical manifestations.

**Table 3:** List of syndrome elements

Group	No	Syndrome element	Group	No	Syndrome element
Eight principle syndrome differentiation (group 1)	1	Yang deficiency	Disease cause syndrome differentiation (group 2)	25	External wind
	2	Yin deficiency		26	Cold
	3	Yang hyperactivity		27	Summerheat
	4	Yang floating		28	Dampness
	5	Exterior		29	Dryness
	6	Half-exterior half-interior		30	Fire-heat
	7	Deficiency		31	Food accumulation
	8	Excess			
Qi–blood–fluid–humor syndrome differentiation (group 3)	9	Qi deficiency	Visceral syndrome differentiation (group 4)	32	Liver
	10	Qi stagnation		33	Gallbladder
	11	Qi sinking		34	Lung
	12	Insecurity of qi		35	Large intestine
	13	Qi counterflow		36	Spleen
	14	Blood deficiency		37	Stomach
	15	Blood stasis		38	Kidney
	16	Blood heat		39	Bladder
	17	Blood cold		40	Heart
	18	Stirring blood		41	Small intestine
	19	Stirring wind		42	Heart spirit
	20	Phlegm		43	Chest and diaphragm
	21	Retained fluid		44	Uterus
	22	Water retention		45	Sinew and bone
	23	Fluid depletion		46	Skin
	24	Essence deficiency		47	Meridian–collateral

#### Clinical evaluation of prototype system

The clinical evaluation of the prototype was conducted at the first affiliated hospital of Guangzhou University of Chinese Medicine in 2013. In May and November, the double blind comparative trial between diagnosis of prototype system and TCM expert was carried out twice. Through the clinical trial, 312 valid medical records were collected. Through comparative analysis of diagnosis of 312 records, the matching results are: there are 171 (54.81%) records whose matched degree is more than 80%, while there are only 6 (1.92%) records whose matched degree is less than 50% [25, 26].

In addition, through the analysis of combination structure of syndrome elements, the structures found from the 312 collected records, are basically identical with the discoveries of Prof. Wang (973 program No. 2003CB517100) [27].

Therefore, it can be concluded that the prototype system of syndrome element measurement is effective in clinical practice.

#### Collection and screening of clinical records

After the clinical evaluation, during the period of 2013–2017, according to the following criteria, the prototype system was used to collect data at school, exhibition, and hospital.

##### Inclusion criteria

(a) People who are willing to detect the health state by the prototype system of syndrome elements measurement; (b) people who can express his feelings clearly; (c) people who can complete the inquiry finally.

##### Exclusion criteria

(a) Records without any symptoms; (b) records whose inquiry time is too much shorter than the normal standard; (c) records whose answers to all inquiry questions are identical.

Finally, including the previous 312 records for clinical trials, 670 (301 males and 369 females) records have been collected for the following analysis.

### Generation of mapping weights

**Fig. 5:**
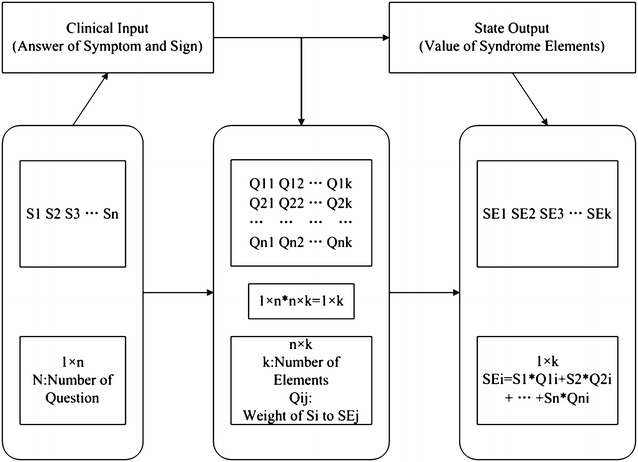
Model of syndrome element quantification

According to the theory of syndrome element differentiation, the quantitative value of syndrome elements can be acquired based on the model shown in Fig. 5. Through the inquiry of system, clinical input data (matrix of answers to symptom-related questions) can be obtained, and then according to the obtained matrix of inquiry answers, the quantitative values of syndrome elements can be acquired based on the matrix of mapping weights.

Therefore, the key to the quantification of syndrome elements is the matrix of mapping weights between clinical input data and syndrome elements. The original matrix of mapping weights of this paper is mainly from Prof. Zhu [16]. Effective as the original mapping data is, there are still several issues unresolved:

- The original mapping data is from statistical analysis of large amounts of clinical records, however it is difficult to explain the meaning of mapping data in the perspective of TCM;
- The original mapping data only gives the mapping weights between set of clinical manifestations and set of syndrome elements, while the mapping relations between other sets of TCM (such as symptoms and formulas) are still uncertain.

In order to resolve these issues, this paper attempts to convert the original mapping weights into condensed ones, which have corresponding meanings in TCM.

Table 4 shows the generation rules of new condensed mapping weights.

**Table 4:** Generation of new weights

Type	Original range (value)	New weight	Type	Original range (ratio)	New weight
Symptom_Normal	[40, 100)	10	Element_Normal	[0.6, 1]	10
	[25, 40)	6		[0.4, 0.6)	6
	[10, 25)	4		[0.2, 0.4)	4
	(0, 10]	2		(0, 0.2)	2
Symptom_Fuzzy	[45, 100)	10	Element_Fuzzy	[0.65, 1]	10
	[40, 45)	8		[0.6, 0.65)	8
	[35, 40)	8		[0.55, 0.6)	8
	[30, 35)	6		[0.45, 0.55)	6
	[25, 30)	5		[0.4, 0.45)	5
	[20, 25)	5		[0.35, 0.4)	5
	[12, 20)	4		[0.25, 0.35)	4
	(10, 12)	3		(0.2, 0.25)	3
	(8, 10]	3		(0.15, 0.2]	3
	(0, 8]	2		(0, 0.15]	2

In the generation of new weights, to explore whether or not the association between different symptoms should be considered, two different generation rules are proposed: *Symptom* and *Element*. Type of *Symptom* refers to the generation of new weights only considers the mapping data of one symptom, while type of *Element* means that the generation of new weights should consider all of the symptoms which are related with one specific syndrome element.

In addition, to explore which granularity level of new weights is more effective, two types of new weights are proposed: *Normal* and *Fuzzy*. *Normal* type means that, according to the mapping correlation compactness between symptom and syndrome element, the new weight data is divided into 4 levels, which are corresponding to the qualitative definition of correlation degree in TCM: 10 (maximum correlation), 2 (minimum correlation), 8 and 6 (medium correlation), while the new weights of *Fuzzy* type are more refined.

Therefore, combining two types of generation methods and two kinds of granularity level of new weights, there are four types of generation rules of new weight: Symptom_Normal, Symptom_Fuzzy, Element_Normal, and Element_Fuzzy.

The new weights generation of Symptom_Normal type is simple:

1. Listing all weight values of one symptom from matrix of original weights;
2. According to the value range of each original weight, generating a new weight value;
3. Integrating the new weight values of the symptom together;
4. Repeating the first three steps, generating new weight values of all symptoms (177 for male and 194 for female);
5. Composing a matrix of new weight values.

Compared with Symptom_Normal type, the new weights generation of Symptom_Fuzzy type is a little complicated:

1. Listing all weight values of one symptom from matrix of original weights;
2. According to the value range of each original weight, generating a new weight value;
3. If the value of original weight is close to the boundary of range, a new secondary weight value (8, 5, and 3) will be generated according to the secondary range;
4. Integrating the new weight values of the symptom together;
5. Repeating the first four steps, generating new weight values of all symptoms (177 for male and 194 for female);
6. Composing a matrix of new weight values.

Compared with type of Symptom_Normal and Symptom_Fuzzy, the new weights generation of Element_Normal type is more complicated:

1. Listing all weight values of one syndrome element from matrix of original weights;
2. Finding the maximum weight value of the syndrome element, and calculating the ratio of each weight value to the maximum value;
3. According to the ratio range of each weight value, generating a new weight value;
4. Integrating the new weight values of the syndrome element together;
5. Repeating the first four steps, generating new weight values of all syndrome elements listed in Table 3;
6. Composing a matrix of new weight values.

Compared with type of Symptom_Normal, Symptom_Fuzzy, Element_Normal, the new weights generation of Element_Fuzzy type is the most complicated:

1. Listing all weight values of one syndrome element from matrix of original weights;
2. Finding the maximum weight value of the syndrome element, and calculating the ratio of each weight value to the maximum value;
3. According to the ratio range of each weight value, generating a new weight value;
4. If the ratio of original weight is close to the boundary of range, a new secondary weight value (8, 5, and 3) will be generated according to the secondary range;
5. Integrating the new weight values of the syndrome element together;
6. Repeating the first five steps, generating new weight values of all syndrome elements listed in Table 3;
7. Composing a matrix of new weight values.

### Evaluation of mapping weights

**Fig. 6:**
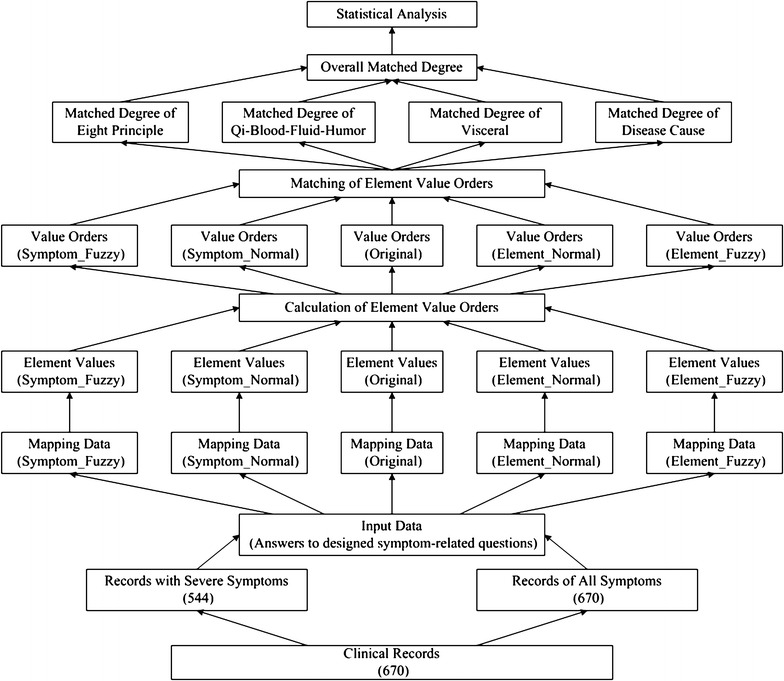
Evaluation model of mapping weights

To evaluate the effectiveness of new weights, the four kinds of new weights will be used to approximate the results of the original mapping data. Figure 6 shows the evaluation model of mapping weights.

The process of evaluation can be divided into several steps:

1. Based on the clinical input data, calculating the quantitative values of syndrome elements according to the corresponding matrix of mapping weights;
2. According to the quantitative values of syndrome element and the group it belongs to, based on specified rules (Table 5), calculating the value order of each syndrome element.

**Table 5:** Rules of value order

Value order	Ratio (value/maxvalue of group)	Weight
8	[0.95, 1]	1
7	[0.9, 0.95)	0.95
6	[0.85, 0.9)	0.9
5	[0.8, 0.85)	0.8
4	[0.6, 0.8)	0.7
3	[0.4, 0.6)	0.6
2	[0.2, 0.4)	0.4
1	(0, 0.2)	0.2
3. Taking the value orders of original weights as standard, calculating the matched degree of each syndrome differentiation group based on the value order of each syndrome element.
  1 $${\text{MDG}} = ( {\text{CG}} - \sum {{\text{SE\_N*SE\_W}}} ) / {\text{CG}}$$
  MDG: matched degree of the syndrome differentiation group; CG: count of syndrome element in the syndrome differentiation group; SE_N: the syndrome element whose value order is not consistent with real value order; SE_W: the weight of syndrome element.
4. Calculating the integrated matched degree according to the matched degree of each syndrome differentiation group.
  2 $$MD = \sum {(MDG*CG/CT)}$$
  MD: matched degree of the integrated syndrome differentiation; MDG: matched degree of specific syndrome differentiation group; CG: count of syndrome element in specific syndrome differentiation group; CT: count of syndrome element in all groups.
5. Statistical analysis of all records according to the matched degree of each record.
6. Evaluation of four kinds of new mapping weights based on the statistical distribution of matched degree.

In addition, for a more obvious contrast, results calculated from all symptoms (*overall health state*) and results acquired from severe symptoms (*major health state*) are evaluated together. As excess (No. 8 element) and deficiency (No. 7 element) are not calculated directly from input answers, so they are excluded during evaluation.

### Knowledge discovery

In this part, APOSD will be adopted to analyze and visualize the combination structure of syndrome elements based on 670 medical records. The process can be divided into several steps:

1. Extracting the syndrome elements whose value order is at the highest level from the 670 collected medical records;
2. Taking syndrome elements extracted from the first step as attributes, and using the medical records as objects, establishing formal context of the 670 medical records;
3. Based on the formal context, generating the corresponding APOSD;
4. Discovering combination structure of syndrome elements from the APOSD.

The knowledge discovered from different types of results will be compared to verify whether they are consistent.

## Results

### Results of weights generation

**Fig. 7:**
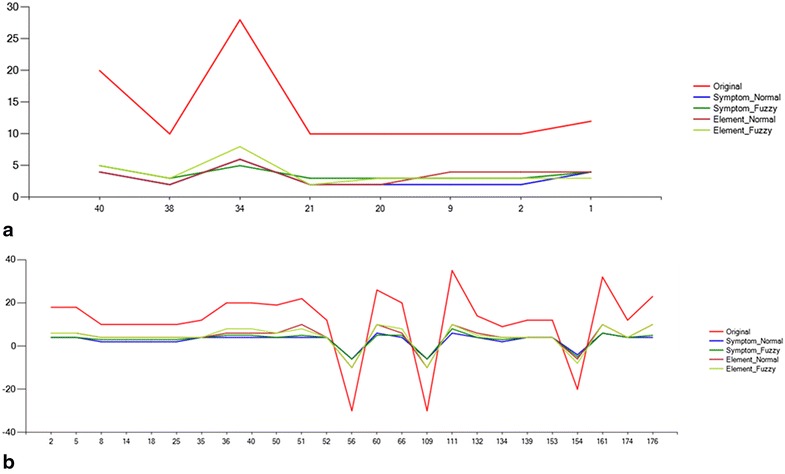
Contrast of mapping weights.** a** Mapping weights of asthma (symptom),** b** mapping weights of yang hyperactivity (element)

Figure 7 shows the contrast examples of mapping weights of different types.

In the mapping figure of asthma (Fig. 7a), the labels of X axis represent the numbers of syndrome elements this symptom is mapping to, while the values of Y axis express the correlation degree of the mapping. In the mapping figure of yang hyperactivity (Fig. 7b), the labels of X axis represent the numbers of symptoms this element is related to, while the values of Y axis express the correlation degree of the relation.

As shown in the figure, despite the weight deviations between different types, the changing trend of the values is consistent, which means that the qualitative mapping relations between medical manifestations and syndrome elements have not changed.

### Results of weights evaluation

**Fig. 8:**
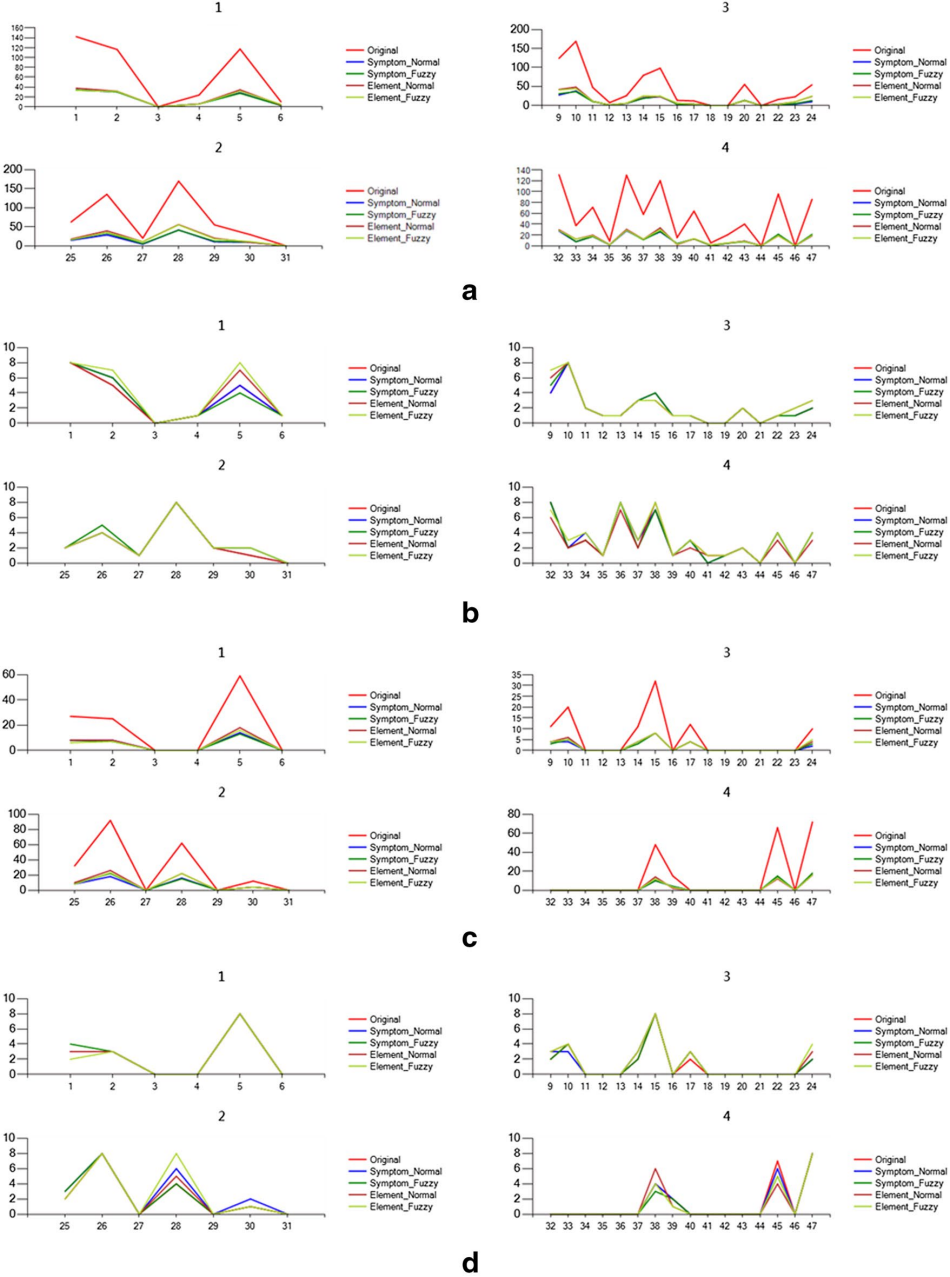
Contrast of values and value orders of syndrome elements.** a** Values of elements calculated from all symptoms,** b** value orders of elements calculated from all symptoms,** c** values of elements calculated from severe symptoms,** d** value orders of elements calculated from severe symptoms

Figure 8 shows the values and value orders contrast of syndrome elements calculated from the input answers of one example record. As shown in the figure, the value orders of the five types are basically consistent, which means that the corresponding qualitative diagnosis results are consistent as well.

Table 6 shows the statistical distribution of matched degree (eight principle, disease cause, qi–blood–fluid–humor, visceral and integrated differentiation) calculated from the 670 records.

**Table 6:** Statistical analysis of matched degree

Group	Matched degree (%)	All samples (670)				Samples with severe symptoms (544)			
		Symptom_Normal	Symptom_Fuzzy	Element_Normal	Element_Fuzzy	Symptom_Normal	Symptom_Fuzzy	Element_Normal	Element_Fuzzy
Eight principle syndrome differentiation	[95, 100]	322	258	167	84	264	265	229	203
	[90, 95)	110	103	112	116	80	84	98	100
	[85, 90)	116	142	83	77	93	94	66	65
	[80, 85)	67	93	114	139	36	38	51	52
	[75, 80)	29	42	98	122	27	29	35	41
	[70, 75)	16	19	58	60	18	16	24	32
	[65, 70)	8	8	20	32	14	9	16	20
	[60, 65)	0	4	12	18	4	1	12	12
	[0, 60)	2	1	6	22	8	8	13	19
Qi–blood–fluid-humour syndrome differentiation	[95, 100]	227	238	24	29	247	248	142	152
	[90, 95)	208	212	86	97	139	156	125	132
	[85, 90)	155	140	144	189	97	85	119	121
	[80, 85)	57	61	172	173	39	35	80	79
	[75, 80)	18	13	124	109	16	14	49	38
	[70, 75)	3	6	86	50	4	5	21	11
	[65, 70)	2	0	25	15	2	1	5	7
	[60, 65)	0	0	6	7	0	0	3	4
	[0, 60)	0	0	3	1	0	0	0	0
Disease cause syndrome differentiation	[95, 100]	318	296	130	92	265	268	195	190
	[90, 95)	173	190	165	139	93	100	121	101
	[85,90)	81	88	159	130	76	62	66	76
	[80, 85)	48	47	94	125	38	46	45	53
	[75, 80)	19	18	48	83	24	35	44	39
	[70, 75)	11	15	39	46	28	18	29	35
	[65, 70)	11	8	15	24	12	5	13	19
	[60, 65)	8	5	11	13	4	4	17	12
	[0, 60)	1	3	9	18	4	6	14	19
Visceral syndrome differentiation	[95, 100]	245	173	118	127	253	240	224	261
	[90, 95)	209	199	163	173	153	146	146	139
	[85, 90)	142	177	172	192	70	90	92	83
	[80, 85)	62	80	109	113	53	52	46	36
	[75, 80)	9	37	70	45	13	13	25	15
	[70, 75)	3	4	30	19	2	3	11	9
	[65, 70)	0	0	5	1	0	0	0	1
	[60, 65)	0	0	2	0	0	0	0	0
	[0, 60)	0	0	1	0	0	0	0	0
Integrated syndrome differentiation	[95, 100]	153	98	4	2	163	169	108	125
	[90, 95)	333	333	102	91	224	233	170	167
	[85, 90)	156	201	258	293	130	117	154	147
	[80, 85)	28	35	224	204	21	22	92	88
	[75, 80)	0	3	70o	70	5	3	17	15
	[70, 75)	0	0	12	10	1	0	3	1
	[65, 70)	0	0	0	0	0	0	0	1
	[0, 65)	0	0	0	0	0	0	0	0

As shown in the Table 6, for the Symptom_Normal type results of 670 all records, the numbers of records whose matched degree is no less than 80% are 615 (91.8% for eight principle group), 647 (96.5% for qi–blood–fluid–humor group), 620 (92.5% for disease cause group), 658 (98.2% for visceral) and 670 (100% for integrated group).

For the Symptom_Normal type results of 544 records with severe symptoms, the numbers of records whose matched degree is no less than 80% are 473 (86.9% for eight principle group), 522 (95.9% for qi–blood–fluid–humor group), 472 (86.7% for disease cause group), 529 (97.2% for visceral) and 538 (98.9% for integrated group).

To sum up, for the integrated syndrome differentiation of 670 records, the matched degree of each record is no less than 65%, while there are at least 87% records whose matched degree is more than 80%.

### Results of knowledge discovery

Among the four new types of mapping weights, the matching result of Symptom_Normal type is the best. Therefore, in this part, the APOSD of Symptom_Normal type is used to compare with the APOSD of original results. In this part, only the annular style of APOSD is adopted. In addition, the outermost circle of objects is removed because of the excessive objects. In the diagrams, the labels are used to represent the syndrome elements (e.g.: ‘***e1***’ represents the syndrome elements whose No. listed in Table 3 is 1).

**Fig. 9:**
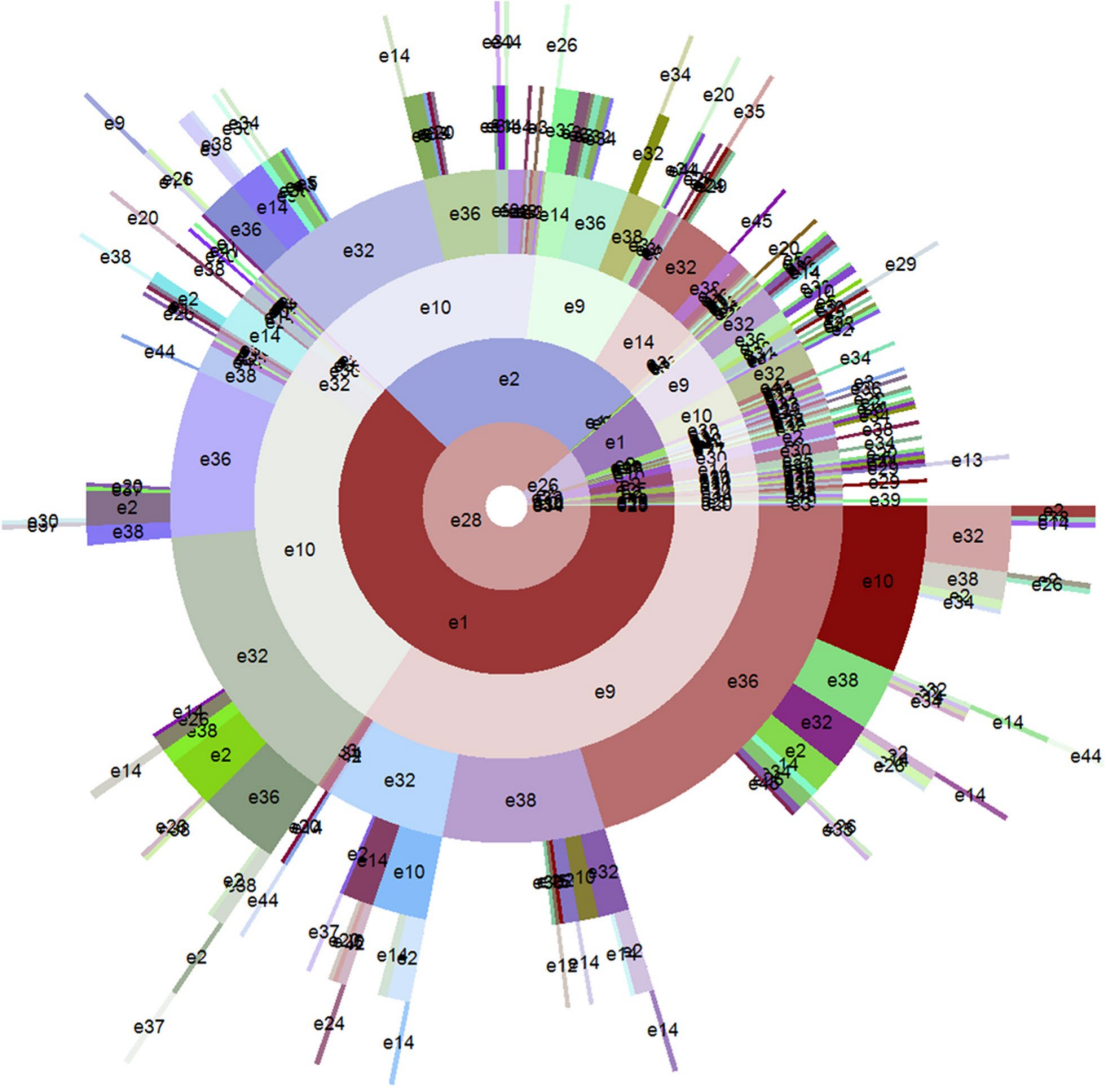
APOSD of original results

Figure 9 shows the hierarchical structure of APOSD generated from the original results of 670 medical records. As shown in the figure, in the innermost layer, {e28 = dampness} is the biggest arc, which means that dampness is the most common syndrome element among the 670 records. Under the arc of {e28 = dampness}, in the second layer, the diagram is divided into two big arcs: {e1 = yang deficiency} and {e2 = yin deficiency}. In the third layer, there are some big arcs for three syndrome elements: {e9 = qi deficiency}, {e10 = qi stagnation} and {e14 = blood deficiency}. In the fourth layer, there are several big arcs for syndrome elements of location: {e36 = spleen}, {e32 = liver} and {e38 = kidney}.

It can be concluded from the APOSD (Fig. 9) that, among the 670 medical records, the most common syndrome elements of location are: spleen, liver and kidney, while the most frequent syndrome elements of nature are: dampness, yang deficiency, yin deficiency, qi deficiency, qi stagnation and blood deficiency.

**Fig. 10:**
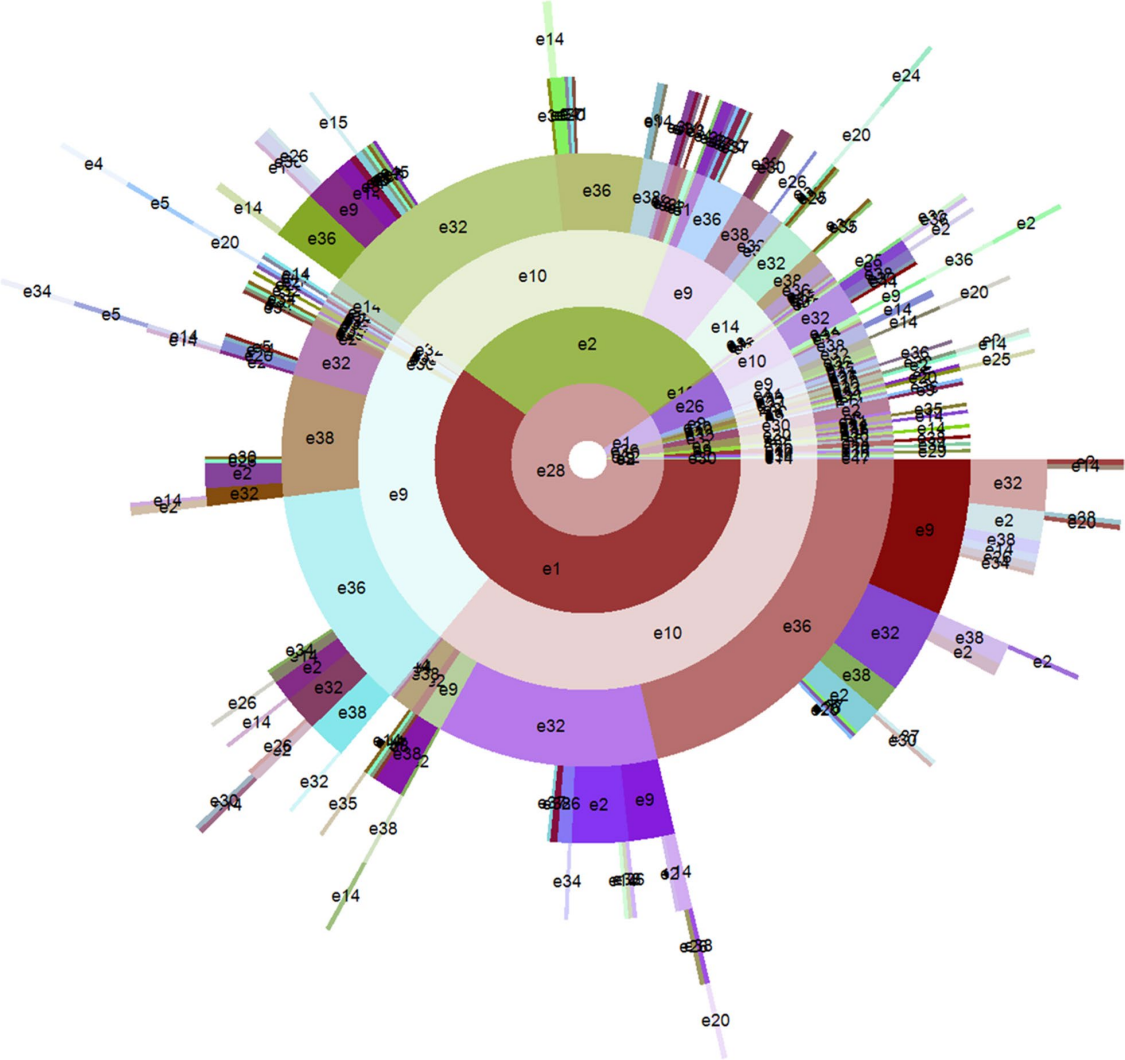
APOSD of Symptom_Normal type results

Figure 10 shows the hierarchical structure of APOSD generated from the Symptom_Normal type results of 670 medical records. As shown in the figure, in the innermost layer, {e28 = dampness} is also the biggest arc. Under the arc of {e28 = dampness}, in the second layer, the diagram is also divided into two big arcs: {e1 = yang deficiency} and {e2 = yin deficiency}. In the third layer, there are also some big arcs for three syndrome elements: {e9 = qi deficiency}, {e10 = qi stagnation} and {e14 = blood deficiency}. In the fourth layer, there are also several big arcs for syndrome elements of location: {e36 = spleen}, {e32 = liver} and {e38 = kidney}.

It can be concluded from the APOSD (Fig. 10) that, among the 670 medical records, the most common syndrome elements of location are: spleen, liver and kidney, while the most frequent syndrome elements of nature are: dampness, yang deficiency, yin deficiency, qi deficiency, qi stagnation and blood deficiency.

From the APOSD, the common combinations of syndrome elements can also be discovered. Table 7 shows the knowledge discovered from the APOSDs of original results (Fig. 9) and Symptom_Normal type results (Fig. 10).

**Table 7:** Results of knowledge discovery

	Original	Symptom_Normal		Original	Symptom_Normal
Common combinations of syndrome elements	e28-e1-e9-e36	e28-e1-e9-e36	Common syndrome elements	Essence	
	e28-e1-e9-e38	e28-e1-e9-e38		Dampness (e28)	Dampness (e28)
	e28-e1-e9-e32	e28-e1-e9-e32		Yang deficiency (e1)	Yang deficiency (e1)
	e28-e1-e10-e32	e28-e1-e10-e32		Yin deficiency (e2)	Yin deficiency (e2)
	e28-e1-e10-e36	e28-e1-e10-e36		Qi deficiency (e9)	Qi deficiency (e9)
	e28-e2-e10-e32	e28-e2-e10-e32		Qi stagnation (e10)	Qi stagnation (e10)
	e28-e2-e10-e36	e28-e2-e10-e36		Blood deficiency (e14)	Blood deficiency (e14)
	e28-e2-e9-e14			Location	
		e28-e2-e9-e38		Spleen (e36)	Spleen (e36)
	e28-e2-e9-e36	e28-e2-e9-e36		Liver (e32)	Liver (e32)
	e28-e2-e14-e32	e28-e2-e14-e32		Kidney (e38)	Kidney (e38)

Through the knowledge discovery, it can be concluded that, despite the differences of details between the APOSDs of original and Symptom_Normal type results, the knowledge discovered from them is basically identical.

## Discussion

In the history of TCM, methods of syndrome differentiation are diverse: eight principle, disease cause, visceral, qi–blood–fluid–humor, six-meridian, triple energizer, and defense-qi-nutrient-blood. These methods provide various cognitions of syndrome from different perspectives. Each of these methods has its own characteristics and scope of application, while all of them are incomplete and need complement of each other. In clinical practice of TCM, the combination of several methods of syndrome differentiation is frequently needed. The coexistence of multiple methods of syndrome differentiation has brought great difficulties to clinical application, teaching and research of TCM.

In view of the above situation, based on the integration of these ancient methods, theory of syndrome element differentiation has been established by Prof. Zhu. Subsequently, in the light of Prof. Zhu’s theory of syndrome element differentiation, based on phenomenology and mathematical mapping, a prototype system of syndrome element measurement has been designed by the team of Prof. Hong. Through clinical evaluation of the prototype system, the mapping data given by Prof. Zhu proved effective. However, the meaning of mapping data is hard to explain in the perspective of TCM and it is difficult to be used for the other sets of TCM.

Therefore, combining phenomenology, mathematical mapping and theory of TCM, four kinds of new mapping weights have been constructed to approximate the results calculated from original mapping data.

**Fig. 11:**
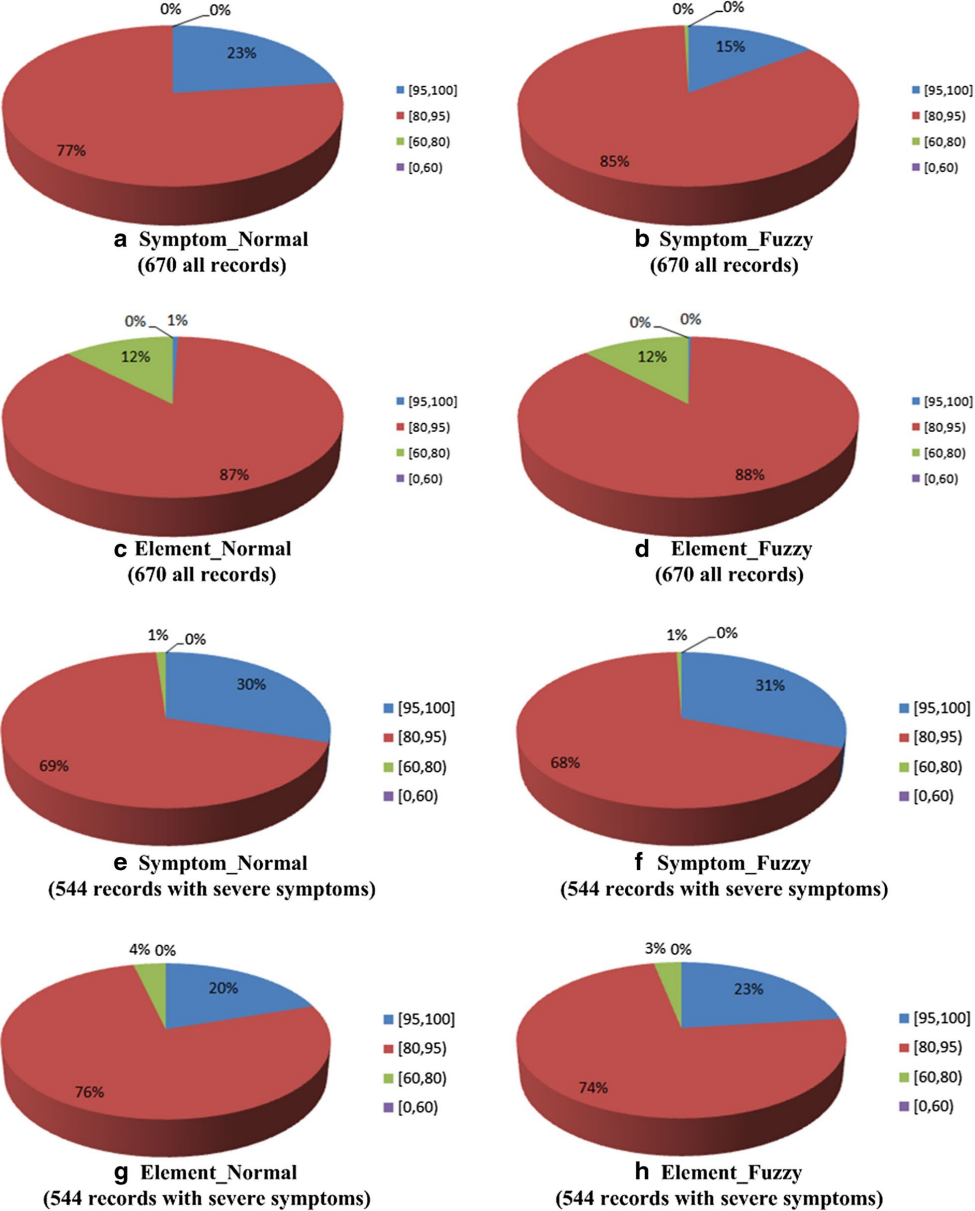
Statistical pies of integrated matched degree

“Results” shows the approximation results of all syndrome differentiation groups, and Fig. 11 shows the statistical pie charts of matched degree under the group of integrated syndrome differentiation.

As shown in the figure, for the Symptom_Normal type results of overall health state (calculated from all symptoms), the matched degrees of all of the 670 records are higher than 80%. For the Symptom_Normal type results of major health state (acquired only from severe symptoms), among the 544 records with severe symptoms, there are 99% records whose matched degree is no less than 80%.

For the results of the other three types (Symptom_Fuzzy, Element_Normal and Element_Fuzzy), the approximation effects are worse than that of Symptom_Normal type.

Therefore, for both overall health state (calculated from all symptoms) and major health state (acquired only from severe symptoms), compared with the approximation results of other three types, the approximation results of Symptom_Normal type are the best. In addition, the matched results of fuzzy types are not higher than that of normal types. Therefore, four levels of mapping weights are already enough, and there is no need to consider smaller granularity.

## Conclusion

In this paper, a new approach to describe and realize the quantitative diagnosis of TCM based on phenomenology is proposed and it is testified through the syndrome element differentiation and knowledge discovery of 670 clinical records. The analyses show that new results of new mapping weights can approximate the results calculated from original mapping data. Therefore, using phenomenology and mathematical mapping to realize the quantitative diagnosis of TCM should be feasible, and mapping data between other sets of TCM can also be determined by the method proposed in this paper.

However, there are still several issues or limitations to be resolved:

1. The evaluation of the new mapping data is mainly based on the research of Prof. Zhu, which is insufficient to some extent. Consequently, further evaluation by clinical experts of TCM should be conducted in future.
2. The original mapping data between clinical manifestations and syndrome elements are static. To achieve self-renewal of the mapping data with the accumulation of medical records, research that using machine learning methods to approximate the mapping relations of TCM should be conducted in future.

## Additional file

**Additional file 1.** Minimum standards of reporting checklist.

## Abbreviations

TCM: traditional Chinese medicine
CWM: conventional Western medicine
APOSD: attribute partial-ordered structure diagram
FCA: formal concept analysis

## References

[1] Feng Y, Wu Z, Zhou X (2006). Knowledge discovery in traditional Chinese medicine: state of the art and perspectives. Artif Intell Med.

[2] Wang H, Cheng Y. A quantitative system for pulse diagnosis in traditional Chinese Medicine. International Conference of the IEEE engineering in medicine and biology society; 2005. p. 5676. 10.1109/IEMBS.2005.1615774 17281544

[3] Wang H, Wang J. A quantitative diagnostic method based on Bayesian networks in traditional Chinese Medicine. International Conference neural information processing, ICONIP, DBLP, Hong Kong, China, October 3–6, 2006; 2006. p. 176–83.

[4] Wang H. A quantitative method for pulse strength classification based on decision tree. International Symposium on information science and engineering. IEEE; 2008. p. 111–15.

[5] Zhao C, Li G, Li F (2014). Qualitative and quantitative analysis for facial complexion in traditional Chinese Medicine. Biomed Res Int.

[6] Li F, Zhao C, Xia Z (2012). Computer-assisted lip diagnosis on Traditional Chinese Medicine using multi-class support vector machines. BMC Complement Altern Med.

[7] Liu G, Li G, Wang Y (2010). Modelling of inquiry diagnosis for coronary heart disease in traditional Chinese medicine by using multi-label learning. BMC Complement Altern Med.

[8] Su S. Recent advances in ZHENG differentiation research in traditional Chinese Medicine. Int J Integr Med. 2013:1. 10.1155/2013/989618 PMC3703351 23853666

[9] Zhao C, Li G, Wang C (2015). Advances in patient classification for traditional Chinese Medicine: a machine learning perspective. Evid-based Complement Altern Med.

[10] Yang X (2015). Chinese traditional medicine: the nature and the roadmap to modernization. Smart Healthc.

[11] Corby D, Taggart L, Cousins W (2015). People with intellectual disability and human science research: a systematic review of phenomenological studies using interviews for data collection. Res Dev Disabil.

[12] Kaivo-Oja J (2017). Towards better participatory processes in technology foresight: how to link participatory foresight research to the methodological machinery of qualitative research and phenomenology. Futures.

[13] Xutian S, Cao D, Wozniak J (2012). Comprehension of the unique characteristics of traditional Chinese medicine. Am J Chin Med.

[14] Wang Y, Xu A (2014). Zheng: a systems biology approach to diagnosis and treatments. Science.

[15] Zhu W (2004). Establishing a new system of syndrome differentiation with syndrome element as core. J Hunan Univ Chin Med.

[16] Zhu W (2008). Study of syndrome elements differentiation.

[17] Hong W, Li S, Yu J (2012). A new approach of generation of structural partial-ordered attribute diagram. ICIC Express Lett Part B: Appl.

[18] Yu J, Hong W, Qiu C (2016). A new approach of attribute partial order structure diagram for word sense disambiguation of english prepositions. Knowl-Based Syst..

[19] Luan J, Hong W, Liu J (2013). The complete definitions of object and abstract description of object features of the formal context. ICIC Express Lett.

[20] Hong W, Sun F, Li S (2016). Partial ordered structure radial tree: a new method for big data visualization. ICIC Express Lett.

[21] Fan F, Hong W, Song J (2016). Visualization method and knowledge discovery of prescription composition. Chin J Biomed Eng.

[22] Fan F, Hong W, Song J (2016). A method of attribute partial-ordered structure diagram for the composition structures of prescription and knowledge discovery. ICIC Express Lett.

[23] Song J, Yu J, Yan E (2013). Syndrome differentiation of six meridians for warm disease based on structural partial-ordered attributes diagram. ICIC Express Lett.

[24] Liu C, Xu S, Li S (2014). Knowledge discovery of formula classification according to therapies in Shanghanlun based on representation method of multi-layer complex concept network. J Beijing Univ Tradit Chin Med.

[25] Luan J. A research about syndrome differentiation assistant system of traditional Chinese medicine based on structural partial-ordered attribute theory. Yanshan University; 2014 (Ph.D. Thesis).

[26] Hong W, Zhang Z, Luan J, et al. A research about value order measurement system of traditional Chinese Medicine syndrome elements. International Conference on medical biometrics. IEEE; 2014. p. 74–9.

[27] Hong W, Song J, Zheng C, Luan J, et al. Comparative study on pattern discovery of traditional Chinese medicine common syndrome elements. International Conference on medical biometrics. IEEE; 2014. p. 68–73.

